# Adaptive and Pathogenic Responses to Stress by Stem Cells during Development

**DOI:** 10.3390/cells1041197

**Published:** 2012-12-10

**Authors:** Ladan Mansouri, Yufen Xie, Daniel A Rappolee

**Affiliations:** 1 Department of Clinical Immunology and Allergy (L2:04), Karolinska University Hospital Solna, Karolinska Institutet, 171 76 Stockholm, Sweden; E-Mail: ladan.mansouri@ki.se; 2 CS Mott Center for Human Growth and Development, Wayne State University School of Medicine, Detroit, MA 48201, USA; E-Mail: yxie@med.wayne.edu; 3 Department of Ob/Gyn, Reproductive Endocrinology and Infertility, Wayne State University School of Medicine, Detroit MI, 48201, USA; 4 Program for Reproductive Sciences and Department of Physiology, Hospital, Wayne State University School of Medicine, Detroit MI, 48201, USA

**Keywords:** embryonic stem cells, trophoblast stem cells, stress, oxidative stress, ER stress/unfolded protein response, genotoxic stress, SAPK/JNK, AMPK, differentiation

## Abstract

Cellular stress is the basis of a dose-dependent continuum of responses leading to adaptive health or pathogenesis. For all cells, stress leads to reduction in macromolecular synthesis by shared pathways and tissue and stress-specific homeostatic mechanisms. For stem cells during embryonic, fetal, and placental development, higher exposures of stress lead to decreased anabolism, macromolecular synthesis and cell proliferation. Coupled with diminished stem cell proliferation is a stress-induced differentiation which generates minimal necessary function by producing more differentiated product/cell. This compensatory differentiation is accompanied by a second strategy to insure organismal survival as multipotent and pluripotent stem cells differentiate into the lineages in their repertoire. During stressed differentiation, the first lineage in the repertoire is increased and later lineages are suppressed, thus prioritized differentiation occurs. Compensatory and prioritized differentiation is regulated by at least two types of stress enzymes. AMP-activated protein kinase (AMPK) which mediates loss of nuclear potency factors and stress-activated protein kinase (SAPK) that does not. SAPK mediates an increase in the first essential lineage and decreases in later lineages in placental stem cells. The clinical significance of compensatory and prioritized differentiation is that stem cell pools are depleted and imbalanced differentiation leads to gestational diseases and long term postnatal pathologies.

## 1. Introduction and Summary of Goals for the Review

Stress is the basis of adaptation and pathology. Thus an understanding of stress gives insight into how cells and organisms remain alive and healthy but may also mortgage their futures as part of the immediate response to stress. This review will analyze basic areas of the stress response with knowledge derived from adult somatic cell responses and the emerging field of stress responses in stem cells of the early conceptus; embryonic and placental trophoblast stem cells (ESC and TSC, respectively). For early stem cells, stress-induced differentiation is an organismal survival strategy in response to stress exposures which limit stem cell population expansion. This leads to more required differentiated function/cell and increases early lineages while suppressing later ones. 

## 2. What Is Stress? General Elements of Cellular Stress

### 2.1. Stressors

Organisms from unicellular prokaryotes to multi-cellular eukaryotes are constantly exposed to conditions that cause acute stress (due to transient environmental changes) or chronic stress (due to long-term fluctuations in maternal or environmental factors). Cells respond to many stresses through changes in metabolic state, secretions and gene expression [1]. These stressors consist of nutrient depletion, depletion of ATP [2], low oxygen condition [3,4], oxidative stress [4], heat shock or hyperthermia, high osmotic exposure [5,6], DNA damage caused by exposure to UV radiation or anticancer therapeutics and nuclear envelope stress ( involving changes in expression level of components and their distribution) [7], cold treatment or hypothermia [8], baric stress [9], heavy metal stress (enhancing cytotoxicity mostly via oxidative stress in addition to their own mechanisms of action) [10], changes of cellular metabolism induced by cancerous transformation [11], pro-inflammatory cytokines [12], mechanical stress [13] and stress hormones [14]. Thus cells are exposed to a wide array of insults and the cellular stress responses are important in regulating cell function or fate. 

### 2.2. Adaptive Responses

A first set of cellular adaptive responses to stress consists of a decrease in almost all macromolecular syntheses; normal proteins, RNA, fatty acids and DNA and increase in synthesis of a group of stress proteins, which is probably regulated by cytoskeletal changes occurring during stress. This phenomenon is highly evolutionarily conserved [15]. The stress proteins act by controlling cell cycle, repairing damaged proteins and folding them back to the native state, regulating gene transcription, antioxidant function and controlling mitogenesis [16]. These adaptive responses include the heat shock protein (HSP) family [17] and the thioredoxin system [18]. It has been claimed that the cells regulate about 44 stress proteins as core mediators out of 368 gene products, known to be evolutionarily conserved among the archaea, the eubacteria and eukaryotes [19,20,21,22] in comparison against the whole human proteome, which reflect different aspects of the stress response; for example, molecular chaperone DnaK (HSP70) and DnaJ (HSP40), MutS/MSH (a component of the eukaryotic mismatch repair machinery), DNA topoisomerase III, Lon (a stress-induced ATP-dependent protease), Glutathione reductase, *etc.* [19,20]. Some regulatory RNAs (micro-RNAs) have emerged that contribute to these mechanisms as well [21,22]. Thus the cellular stress response is a complex mechanism set which involves a variety of cellular functions. 

Most members of the HSP family are synthesized under normal conditions of growth before stress. We call this stress response pathway a cellular/organismal “health insurance policy”, as health insurance (stress response mechanisms) are produced while cells are healthy and replete with energy before stress. Obviously the molecules synthesized prior to the stress response do not just sit around in a static state before stress. They are involved in cell proliferation, signal transduction, anti-apoptotic functions, growth factor and cytokine-like effects [23] and also protein folding, assembly, translocation, and degradation [24]. This indicates that these proteins regulate physiological functions as well as cell stress responses.

**Figure 1:**
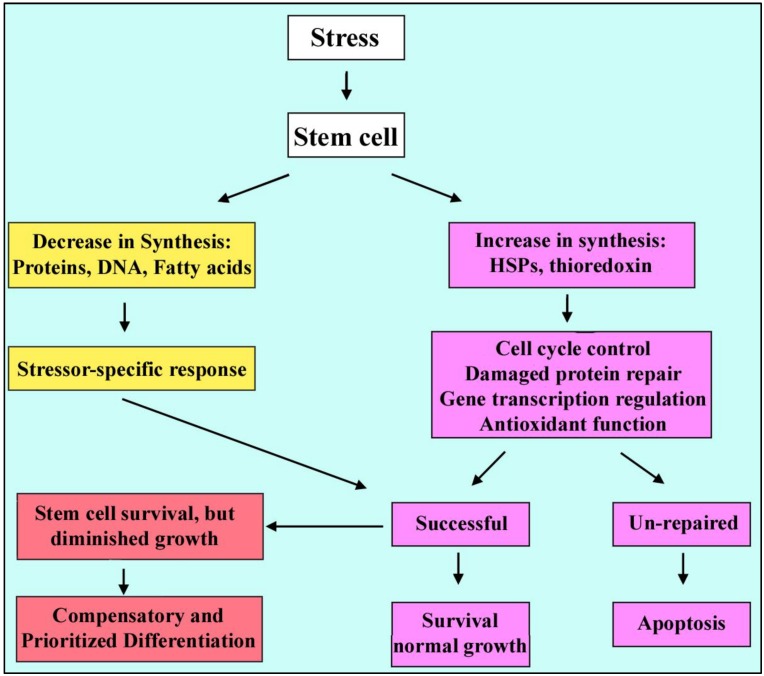
Stem cell adaptive response. Cell will respond to stressors in different ways, through reducing the macromolecular production and increasing the heat shock protein synthesis, in parallel with some stressor-specific responses. These adaptations are in favor of cell survival otherwise the apoptotic pathways become activated, leading to cell death.

A second set of adaptive responses (cellular homeostasis responses) initiate during cellular stress. This set of responses is stressor-specific with a slower onset, which is involved in re-establishing homeostasis when healthy cells survive the initial period of stress, and continue until conditions change again. For example, during exposure to hypertonic stress, transporters become activated and enzymes function more, so that they control the accumulation of compatible organic tissue– specific osmolytes to counterbalance extracellular hypertonicity [19,25]. Thus cell responses to environmental stressors can be considered as homeostasis responses specific to those stressors but which incorporate signaling pathways that are shared between stressors (Figure 1).

### 2.3. Stress Outcomes

The changes initiated by stress may last for hours to days according to the effect of stress on the biochemical environment and gene expression [26]. Stress may result in epigenetic modifications (DNA methylation and histone methylation, phosphorylation and acetylation), which change the expression of the genes without altering the DNA, or genomic changes may occur due to single-strand or double-strand breaks and structural rearrangements [27]. These alterations can be passed on to daughter cells in the cell lineage and some groups of proteins such as Polycomb-group (PcG) and Trithorax-group proteins (trxG) are, respectively, involved in remembering and maintaining the silent or active pattern of expression of associated genes [28]. Thus DNA-damaging agents, and other stressors, may lead to some changes which can be maintained in many cell generations.

Stress response initiates by upregulation of HSPs mediating refolding of damaged proteins as well as maintaining genomic integrity via nucleotide excision repair (NER) [29,30,31]. If anti-stress mechanisms are not successful, intracellular proteins become denatured and insoluble, tending to aggregation and precipitation. Denatured proteins are not functional and must be eliminated. When a protein is un-repairable, a signal can be sent to the HSPs by some modiﬁcations such as carbonylation (irreversible protein oxidation). The HSPs which are ubiquitin-dependent proteases will cause degradation of the damaged proteins. There are mediating proteins that can switch adaptive responses to apoptosis *i.e.* in DNA damage response, ataxia telangiectasia mutated (ATM) is recruited to sites of broken DNA and phosphorylates checkpoint kinase 2 (Chk2) and subsequently p53. In sub-lethal damages, survival pathways are engaged via p21-mediated cell cycle arrest but if the damage is too severe pro-apoptotic p53 target genes become activated which promote apoptosis [32]. So cells can respond to stress, depending on the level and mode of stress, in various ways ranging from the activation of survival pathways and adaptation in mild stresses to the initiation of cell death in the severe stresses.

### 2.4. Stressed Stem Cells

There are different types of cell in normal adult tissues which are organized in a hierarchal manner to sustain tissue integrity and function. Multipotent adult stem cells locate at the top of the hierarchy and give rise to progenitor cells and differentiated cell types with specific functions [33]. These cells respond differently to the same exposure due to varying state of differentiation and degree of protection and repair [34]. Pluripotent embryonic stem cells differentiate into all tissues of the embryo and have highly efficient mechanisms to repair any DNA damage resulted from stressors. Stem cells are proficient in antioxidant defense but several antioxidant and stress-resistance genes become down-regulated during early steps of differentiation into embryoid bodies [35]. Oct4 (octamer-binding transcription factor 4), also known as POU5F, is necessary for the maintenance of the embryonic stem cell phenotype and pluripotency (through splicing variant Oct4A) as well as stress resistance and response pathways (through splicing variant Oct4B) [36,37,38]. The expression of this transcription factor is also down-regulated during differentiation of stem cells [39]. 

Stem cells in the early embryo may give rise to large clones of mutant cells if DNA damage is not repaired. Interestingly stem cells in the irradiated blastocyst attempt DNA repair, but the most rapidly proliferating stem cells in gastrulation undergo apoptosis at the same irradiation dose [40]. There is a lack of a G1 checkpoint in embryonic stem cells so that mutated cells can progress into S-phase, where the damage can be exacerbated, and this leads to cell death [39,41] Thus stem cells in the embryo have enhanced mechanisms to prevent and repair DNA damage or kill damaged stem cells and they are different from somatic cells in terms of their capacity to repair or alternatively to lead in apoptosis.

**Figure 2:**
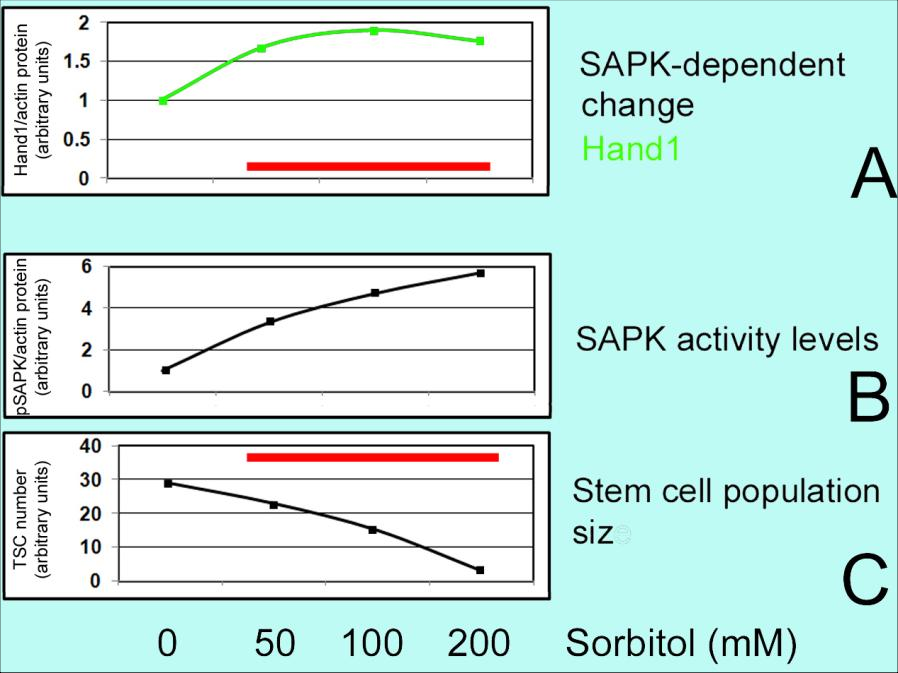
S-shaped curve for compensatory and prioritized differentiation with a reverse S-shaped curve for stem cell accumulation. As the dose of hyperosmolar stress (sorbitol) increases, the stem cell accumulation rate decreases (C.) but the stress enzyme (SAPK) activity level (B) and differentiation marker (Hand 1) (A) level rise. Standard error of the mean was between 1%–8% of the mean. Red bars in (A) and (C) show range of stress-induced differentiation.

We discussed two types of stress response, decreased macromolecular synthesis and hormesis of tissue- and stress type-specific stress responses that rescue the stressed cell. During development, pluripotent and multipotent stem cells have a third type of stress response. They differentiate when stress diminishes stem cell population expansion, and they differentiate to earliest lineage while suppressing later lineages. We found that hyperosmolar stress causes dose-dependent and time-dependent changes in embryo growth and stem cell accumulation as well as apoptosis, as shown in Figure 2, e.g., 200 mM dose of sorbitol caused stasis of placental trophoblast stem cell (TSC) population size expansion and cell accumulation [42,43]. The stress enzyme stress-activated protein kinase (SAPK) is activated over a long dose range in a sigmoidal dose response for sorbitol [5] and other stressors [3,44,45]. When this occurs SAPK-dependent differentiation is mediated by heart and mesoderm inducer (Hand1) transcription factor [46]. As stem cell accumulation diminishes, SAPK-dependent differentiation increases. Hand1 normally mediates induction of the first TSC-derived lineage after the embryo implants into the uterus but stress rapidly induces Hand1 and the Hand1-dependent placental lactogen (PL) 1 protein markers of this first lineage. The induction of the first lineage is not limited to PL1 but a global analysis shows that many markers of the first lineages are induced by 24 h of 400 mM sorbitol [6]. The early global response in the first 30 minutes is accompanied by decreased mRNA synthesis, in agreement with the known early stress-induced decreases in macromolecular synthesis.

Cultured in 400 mM sorbitol, TSC mounted a massive differentiation to the first lineage but lost the ability to recover between 4 and 24 h. Embryos resisted 1,000 mM sorbitol for more than a day before their demise [43] whereas stem cells die within hours of addition of 1,000 mM. Time- and dose-dependent responses to stress, cell cycle arrest, apoptosis and SAPK induction and role are similar in stem cells and somatic cells but embryos can tolerate particular levels of hyperosmolar stress for days while somatic cells may go to die within a few hours of exposure.

There are two unique properties which belong to stem cells of the embryo and placenta known as ''compensatory differentiation'' and ''prioritized differentiation'' [47]. When stress occurs at low doses, stem cell accumulation rates are not significantly diminished and apoptosis is not significantly increased and stem cells will survive by using adaptive mechanisms, but at high doses of stress, stem cells cannot proliferate sufficiently and stress induces a special kind of differentiation to fulfill an essential function in development (e.g. differentiation markers such as Hand1 and PL1) [6,44,46], as shown in Figure 2. Moreover, stress at lesser exposures induces only stem cell survival but greater exposures that diminish stem cell accumulation rates also induce organismal survival through tightly linked compensatory and prioritized differentiation of stem cells. Thus there is a threshold where stem cells transition from cellular survival to organismal survival and prioritized differentiation allows stem cells to adapt to stress through induction of early essential differentiated lineages and suppression of later essential differentiated lineages [48].

**Figure 3:**
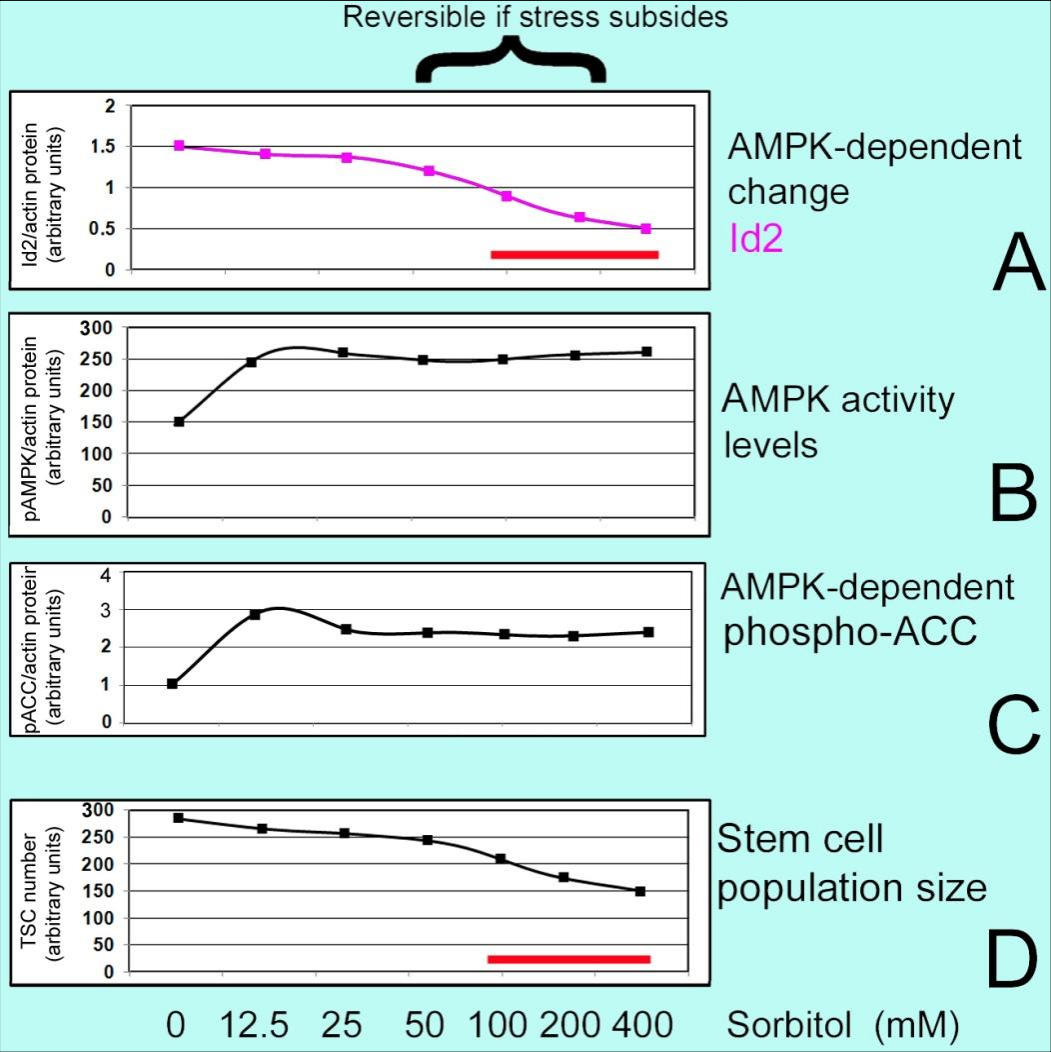
S-shaped curve for low dose stem cell survival and high dose organismal survival through compensatory differentiation. At low concentrations of Sorbitol, stem cell population size (D) and Inhibitor of differentiation (ID2) level (A) are not significantly changed but AMPK-dependent phosphorylation of ACC and inactivation of its anabolic activity do occur at low doses (C). At higher doses the accumulation rate decreases (D) and AMPK-dependent (B) decrease in Inhibitor of differentiation (ID2) protein level leads to induction of differentiation. Standard error of the mean was between 1%–8% of the mean. Red bars in (A) and (D) show range of stress-induced differentiation.

A specific example of compensatory differentiation occurs when hyperosmolar stress induces AMP-activated (AMPK) stress enzyme-dependent phosphorylation and inactivation of the anabolic enzyme acetyl CoA carboxylase (ACC, a product of the *ACACA* gene) at low hyperosmolar sorbitol doses (Figure 3). But only at higher stress doses does AMPK mediate loss of Inhibitor of Differentiation (ID) 2 protein. It is at this higher dose range of stress that TSC population expansion is significantly diminished. ID2 is necessary during normal development as ID2 blocks Hand1-dependent PL1 induction. Thus higher levels of stress induce AMPK-dependent loss of ID2 and SAPK-dependent gain of Hand1 and Hand1-dependent PL1 induction when stem cells populations don’t expand normally. 

## 3. Effects of Oxidative Stress on Stem Cells

### 3.1. Reactive Oxygen Species

Our body is constantly stimulated by reactive oxygen species (ROS) which are either produced by energy–deriving oxidative metabolism endogenously or following exposure to the chemicals, low-level radiation and other insults [33]. In most cells, under most conditions, the mitochondria, ER, and peroxisomes are the major source of ROS [49,50]. ROS can be involved in physiological functions such as regulation of vascular tone, ventilation, cell adhesion, immune responses, programmed cell death and signal transduction from membrane receptors [51]. On the other hand ROS can damage all major cellular constituents at high concentrations [51,52]. An antioxidant defense system has evolved that holds these in balance. However, this balance can be perturbed and result in oxidative stress. 

### 3.2. Oxidative Stress Outcomes

Minor disturbances lead to homeostatic adaptations whereas major perturbations may lead to damage and cell death through apoptosis or necrosis. Under conditions of stress, nuclear factor kappa-light-chain-enhancer of activated B cells (NF-κB) becomes activated, translocates to the nucleus and induces expression of pro-inflammatory molecules and cytokines [53]. The cells may respond to stress through a family of mitogen-activated protein kinases (MAPK), p38 MAPK and stress-activated protein kinase–c-Jun amino terminal kinases (SAPK–JNK) which regulate cell proliferation, differentiation, apoptosis, migration and cytoskeletal integrity. ROS causes lipid peroxidation in the plasma membrane (associated with loss of fluidity and function, activation of the apoptotic cascade), protein oxidation (led to abnormal protein folding, protein aggregation) and cell death. ROS imbalances may lead to disturbance of intracellular Ca2+ homeostasis, which maintains the function of protein-folding machinery, and the accumulation of misfolded proteins within the lumen of endoplasmic reticulum [54,55]. Also DNA can be attacked and mutated or aberrant gene expression may result. There are some interactions between oxidative and other forms of cell stress, such as endoplasmic reticulum (ER) stress [56]. So oxidative stress induces various cellular responses depending upon the severity of the insult and the compartment in which the ROS are generated [19].

### 3.3. Stressed Stem Cells

Throughout the lifetime of the organism, pluripotent or multipotent stem cells divide and expand the pool through self-renewal as well as giving rise to differentiated mature cells. They are exposed to various stressors over a long period of time such as oxidative stress and ROS. As mentioned earlier, the antioxidant system and adaptations; residing in areas of low oxygen and relying on glycolysis rather than mitochondrial metabolism [57], result in protection of the stem cells from oxidative damage [4]. These properties help the stem cells maintain their full pluripotency otherwise they tend to undergo apoptosis or differentiation (in embryonic and trophoblast stem cells) [48]. In summary, stem cells often reside in a low O_2_ niche (the lower end of a 1%–9% range, depending on the specific environment and tissue) which maintains a slow cycling stage and reduction in mitochondrial respiration and ROS accumulation. This slow-cycling proliferation is mentioned with regard to the hematopoietic stem cells. It has been proposed that average O2 tension is very low in BM blood and in such areas the proliferation of stem cells is almost blocked (as mitochondrial respiration seems to be essential for entering a mitotic cycle) but growth factors sustain long-term maintenance of stem cells through stimulation of glycolysis and better oxygenated areas would allow proliferation of more differentiated progenitors. Hypoxia promotes “stemness” through regulation of factors and molecules involved in differentiation of stem cells [58,59,60,61], e.g., expression of Oct-4 is essential for maintenance of the undifferentiated state and it will be down-regulated when stem cells differentiate. Oct4 is sensitive to oxidation and under oxidative stress conditions, its DNA-binding ability changes. An important regulator of the redox state of this transcription factor is Thioredoxin (Txn) which restores the DNA binding capability of Oct4. It has been shown that an oxidizing agent (e.g., Diamide) can abolish the DNA-binding ability of Oct4 but by increasing the concentration of pure recombinant Txn, this effect can be reversed and the Oct4 binding ability will be restored. Thioredoxin is necessary for full Oct4 activity and without that, even small amounts of oxygen will disrupt its activity (e.g., preventing the early embryonic development and inner cell mass to form proper lineages) [62,63]. 

Txn knockout mice have been studied and Txn (-/-) embryos showed defects in trophoblast outgrowths, hatching and proliferation of Inner cell mass (ICM), *in vitro*. Homozygosity for the Txn null mutation resulted in lethality at the peri-implantation stage. The failure of Txn activity in knockout mice probably makes the ICM cells more vulnerable to ROS and this contributes to inability of ICM cells to proliferate despite of trophoblast-induced decidual reaction [64,65]. Under *in vitro* conditions, the concentration of oxygen is higher than that *in vivo* which results in retarded growth and developmental blockage of embryos due to damage to embryonic cells by oxygen radicals while it’s not seen *in vivo*. It has been observed that Txn promotes the development of mouse embryos *in vitro*. By increasing the concentration of Txn in the culture media (50 µg/mL thioredoxin 4.2 µmol/mL), the number of embryos at the four-cell and blastocyst stage increased significantly, compared with culture in basic medium, and then decreased gradually at higher concentrations [66]. Thus, Txn plays an important role in cell viability, embryo growth and development during peri-implantation embryonic development.

#### 3.3.1. Optimal O_2_ Level in Cultures

The study of Hematopoietic stem cells (HSC) has shown that ROS has no effect on the number of progenitors but inhibits the repopulating of stem cells [67]. In addition to loss of the self-renewal ability, HSCs are also prone to genomic alterations and significant chromosomal instability (nearly 50% aneuploid cells after 2 day *in vitro* culture under normoxic conditions, 20% O_2_) [68]. So optimization of culture conditions by controlling the oxygen levels should be considered in *ex vivo* expansions of HSCs, as well as ESCs, and bone-marrow derived mesenchymal stem cells (MSCs) [69,70].

**Figure 4:**
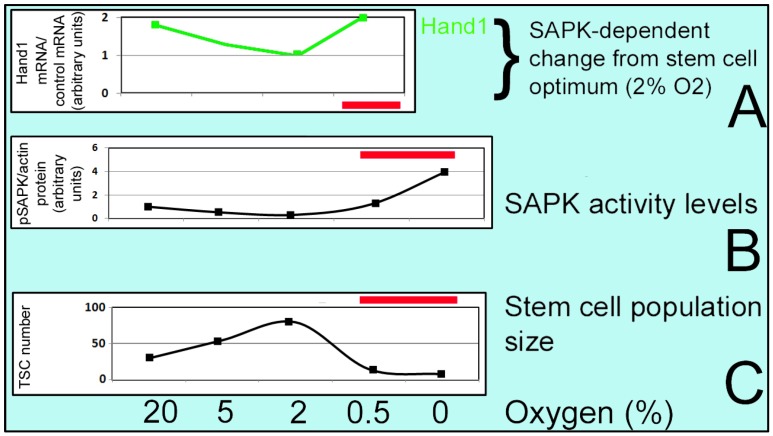
U shaped curve for prioritized differentiation. At 2% O2, Cell accumulation rate is highest and SAPK activity is lowest. At 0–0.5% O2, the accumulation rate is lowest and SAPK activity is highest. As O2 moves from 2%, SAPK leads to increase in earlier lineages (through Hand1) and to decrease in later lineages. Standard error of the mean was between 1%–8% of the mean. Red bars in (A), (B), and (C) show range of stress-induced differentiation.

It has been shown that placental trophoblast stem cells (TSC) are cultured optimally at 2% O_2_ [4] with fibroblast growth factor (FGF) 4 present. FGF4 is synthesized in the inner cell mass of the blastocyst [71] and is necessary to maintain adjacent polar trophectoderm TSC and TSC in culture [4,72,73]. At 2% O_2_ cell accumulation is high, pSAPK activity is low, multipotency markers are high and differentiation markers; e.g., heart and mesoderm inducer transcription factor (Hand1) are low, as shown in Figure 4. 

As in Figure 2 where hyperosmolar stress induces an S-shaped response and a reverse S-shaped curve for growth, SAPK activity is inversely proportional to growth rate. Highest growth and potency are associated with lowest stress and the optimal conditions for the stem cell program. As the milieu deviates from the optimum SAPK activity and SAPK-mediated prioritized differentiation increases. 

At 20% O_2,_ accumulation rate and multipotency markers are lower but pSAPK activity and differentiation markers are higher. Accumulation rate and multipotency markers are lowest at 0–0.5% O_2_, whereas pSAPK activity and differentiation markers are highest and there is a change in balance moving from multipotency toward compensatory differentiation [3,4]. The TSC response to incorrect oxygen stress displays a biphasic or U-shaped curve which is termed hormesis (a low-dose stimulation/ high-dose inhibition manner) [23,24,25]. As mentioned above, at 2% O_2_ cell accumulation and multipotency markers are much higher for TSC compared with either 20% O_2_ or 0–0.5% (0.5% O_2_ will continue to favor differentiation). Stressors force the system to work harder and maintain the cellular homeostasis (low-dose stimulation), even may lead to overcorrection, but at higher doses of stress, this system is unable to respond properly because of an overload on it. This may be due to high-dose inhibition differentiation through increased potency or mitochondrial insufficiency at low O_2_ levels.

#### 3.3.2. ATP Production in Stem Cells

Mitochondrial oxidative phosphorylation (OxPhos), via the electron transport chain (ETC), is the main ATP and energy source in the cells and ROS are respiration by-products. It is shown that in the pluripotent stem cells (PSC), generation of ATP mainly relies on glycolysis rather than OxPhos in differentiated cells [74]. The prevention of glucose oxidation and glycolysis promotion in PSC is regulated by Uncoupling protein 2 (UCP2) which suppresses the channeling of glycolytic flux into the Krebs cycle and decouples respiration from ATP synthesis, attenuates ROS accumulation, maintains genomic integrity and cell viability. UCP2 is significantly higher in PSCs than in differentiated cells and will be repressed by stimulation of cells to differentiate which is necessary for shifting from glycolysis to mitochondrial OxPhos in parallel with ROS accumulation [75]. 

#### 3.3.3. Diverse Adaptive Responses and Pathways in HSC and ESC

It has been claimed that elevation of ROS increases expression of the cell cycle inhibitors p16^Ink4a^ and p19^Arf^, which induce loss of the HSC compartment, and this is not the case for the differentiated cells [67]. This up-regulation is managed by p38 MAPK that limits the lifespan of HSCs [76]. ROS can also induce SAPK/JNK-dependent altered differentiation of hematopoietic stem cells in drosophila [77], mediated by suppression of polycombs and PRC2 mediated promoter methylation. Similarly SAPK also mediates a necessary suppression of polycombs in wounding stress-induced altered methylation in drosophila imaginal disk stem cells [78].

Ataxia Telangiectasia Mutated (ATM) gene, which encodes for a large serine/threonine protein kinase, is involved in the self-renewal of human stem cells through suppression of ROS generation. Atm kinase becomes activated by DNA damage and contributes to genomic stability by phosphorylation of effector molecules [67,79]. In Atm-deficient mice, early-onset bone marrow failure will develop due to functional defect in HSCs but not in differentiated cells. Elevated ROS results in DNA damage and genomic instability in Atm-deﬁcient HSCs as well as activation of p38 MAPK which induces the cell cycle inhibitors p16 ^Ink4a^ and p19^Arf ^and slows self-renewal [80]. Atm is one of the genes that are expressed less in Oct1 deficient cells. Oct1 is widely expressed in adult and embryonic tissues and shares a significant homology with Oct4. Atm contains near-perfect octamer sequence which is an Oct1 binding site and can be regulated via this protein under stress conditions. Oct1-deficient fibroblasts are hypersensitive to stressors such as γ-radiation which can be due to misexpression of Oct1 target genes [81,82]. It is likely that Oct4 function in pluripotent stem cells is similar to Oct1 function in adult somatic cells. 

The forkhead O (FoxO) family of transcription factors upregulates the genes involved in detoxification of ROS and ROS effects, such as Magnesium superoxide dismutase (MnSOD), Catalase and Growth arrest and DNA damage induced proteins (Gadd45), This protects the hematopoietic stem cells from oxidative stress. Following DNA damage, expression of the Gadd45 protein is induced which modifies DNA accessibility on damaged chromatin and interacts with Proliferating cell nuclear antigen (PCNA), a protein involved in DNA replication and repair [83]. It has been shown that Oxidative stress activates the Gadd45 promoter in a FoxO-dependent manner [84]. Loss of FoxO3 in Foxo3a^−/−^ mice is correlated with decreased levels of SOD, catalase and increased p38MAPK activation so that stem cell function is impaired in FoxO-deficient animals due to ROS accumulation and p38 MAPK activation [85,86]. Thus FoxOs may take part in modulating the survival or properties such as self- renewal and differentiation of stem cells.

**Figure 5:**
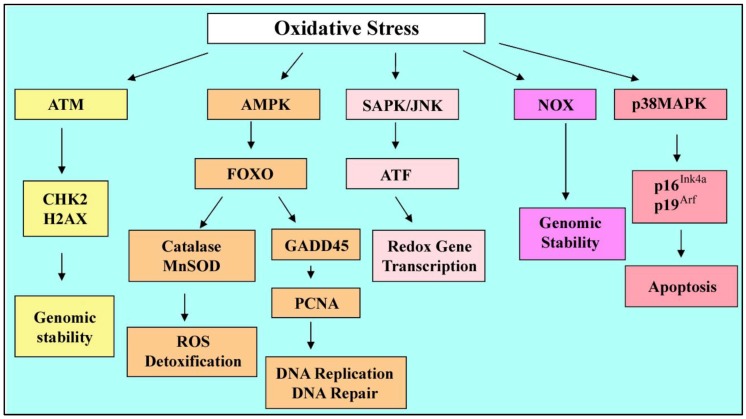
Oxidative stress and adaptive responses. Genomic stability mediated by ATM is promoted through activation of cell cycle inhibitor checkpoint kinase 2 (Chk2) and DNA damage response protein and histone variant H2AX. FoxO family regulates ROS detoxification and DNA repair by Catalase, MnSOD and GADD45 that lead to cell survival. Under stress conditions, the NOX enzyme may be induced and result in increased ROS production and genomic instability. Under higher levels of ROS, cycle inhibitors p16^Ink4a^ and p19^Arf^ may become upregulated through p38 MAPK which promote apoptosis of damaged cells.

Ionizing radiation induces oxidative stress in HSCs and may lead to genomic instability in the descendants of irradiated HSC as well as neighboring un-irradiated stem cells which receive the ROS signals. Studies have shown that characteristic of radiation responses are also seen in non irradiated cells (descendants of irradiated cells due to genomic instability and neighboring unirradiated cells due to communication between these cells, receiving signals which are called radiation-induced bystander effects. It was shown that macrophages are the source of the signals, mediating the bystander effect and TNF-α signaling is implicated in the mechanism) [87]. Increased production of ROS is attributed to the increased function of mitochondria [88] and also through activation and induction of Nicotinamide adenine dinucleotide phosphate oxidase (NOX). Thus exposure of stem cells to oxidative stress coupled with up regulation of NOX results in increased production of ROS and mediates genomic instability (Figure 5) [89]. 

There are high levels of p53 expression in undifferentiated ESC which result in apoptosis in response to oxidative stress, but differentiated cells have lower levels of p53 and are relatively stress resistant [90]. In the absence of p53, the surviving stem cells may carry cellular damages such as DNA breaks and pass it on to the differentiated cells. So this damage (which normally must trigger the p53 dependent pathway) may initiate delayed p53-independent mechanisms (e.g., other p53 family members such as p63, p73) to limit long term survival of damaged cells [91]. It has been shown that in p53-/- embryos, the p53 independent pathways are responsible for preventing persistent genetic lesions in differentiated cells.

In summary, oxidative stress can change the fate of stem cells. At low doses of ROS, stem cells can adapt and the cellular stress response may even lead to overcompensation so that the cells become more resistant to the following insults. But at higher doses of stress, the antioxidant system may become overwhelmed and the cells go under apoptosis. Stem cell niches are microenvironments with low level of O_2_ and decreased level of ROS exposure. After exposure to different doses of O_2_, we can see a dose-response relationship which is displayed in a U-shaped curve known as hormesis. There is a variety of regulatory proteins which are involved in cellular homeostasis insulted by oxidative stress such as Oct4, ATM, FoxO, GADD45, *etc.* We know that the embryonic stem cells and trophoblast stem cells react differently and when they are insulted by the stressor, the population expansion decreases and the multipotency markers decrease. On the other hand there will be a transition from cellular survival to organismal survival and the stem cells adapt through compensatory and prioritized differentiation which induces essential early lineages and suppress later lineages [48].

## 4. Effects of ER Stress on Stem Cells. Prioritized Differentiation is an Adaptive Response

### 4.1. The Endoplasmic Reticulum and Stress

**Figure 6:**
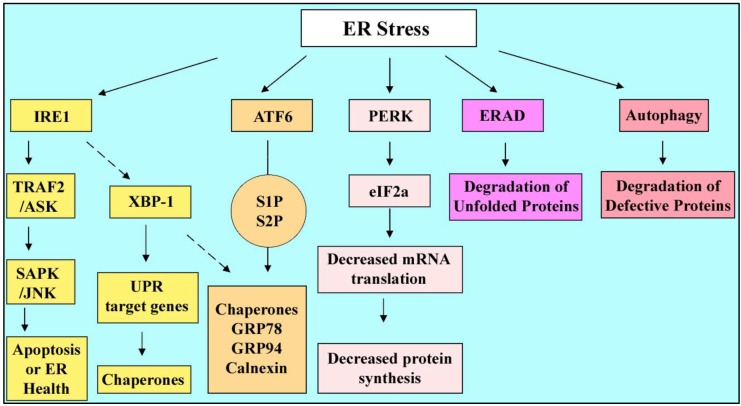
ER stress responses. Different signaling pathways are involved which promote production of chaperones to fold back the unfolded proteins ( IRE1, ATF6) or reduction of protein synthesis (PERK) to decrease the work load on ER. ERAD and autophagy also cooperate with these mechanisms to maintain the balance.

The endoplasmic reticulum (ER) plays essential roles in multiple cellular processes such as Calcium homeostasis, lipid biosynthesis, folding and post-translational modification of proteins (facilitated by molecular chaperones and a variety of enzymes). ER stress occurs due to disturbances by oxidants or reducing agents, hypoxia, glucose deprivation (interfering with N-linked protein glycosylation), disturbances of Ca2+ regulation (impairing the functions of Ca2+-dependent ER chaperones), viral infections and high fat diet [92,93], resulting in an imbalance between the load of proteins facing the ER and the ability of this organelle to process them (Figure 6). Thus ER handles a certain load of proteins, set by developmental and physiological demands. Under stress conditions, ER is not able to do this properly and this causes protein unfolding and misfolding due to lack of disulfide bonding or glycosylation, ineffective chaperones and other disturbances.

### 4.2. ER Stress Response Pathways

The ER stress response involves three main components. First, ER stress can promote upregulation of the genes encoding ER chaperones. This process is called Unfolded Protein Response (UPR). IRE1 is the central enzyme in this type of response (UPR) which is composed of a luminal domain which sense misfolded protein overload in ER and a cytoplasmic portion with kinase and endoribonuclease activity. Following the stress, RNase activity of the cytoplasmic domain results in X-box-binding protein-1 (XBP-1) mRNA splicing and produces a competent XBP1 protein that increases transcription of genes that augment UPR target genes encoding for chaperones [94,95]. Thus this pathway results in production of components which increase ER protein-folding capacity. On the other hand, it has been shown that there are ER-targeted mRNAs (encoding plasma membrane and other secreted proteins) whose repression is dependent on IRE1 but not on XBP-1. These proteins traffic through the ER (not involved in ER function) and IRE1-dependent repression of these proteins is well-suited to relieve ER stress by preventing the translation of proteins targeted to the ER [96].

Second, cells may adapt to ER stress through repression of protein biosynthesis. A kinase called pancreatic ER kinase or PKR-like kinase (PERK), also composed of a luminal region and a cytoplasmic domain, can sense the presence of unfolded proteins through the luminal part and links the stress to the eukaryotic initiation factor2a (eIF2a) phosphorylation, through kinase activity of cytoplasmic domain, which in turn will decrease mRNA translation and protein synthesis [97,98]. This pathway leads to a decrease in production and load of proteins facing ER during the response to existing unfolded proteins. In addition eIF2a phosphorylation leads to transition of mRNA from ribosomes to stress granules that are marked by HuR. This is an important feature of many stress responses besides the UPR, and insures that the high synthetic cost is avoided that would come from destroying and then re-synthesizing mRNA during stress and stress reversal.

Activating Transcription Factor 6 (ATF6) is the third component. Accumulation of unfolded proteins in the ER, results in relocation of this membrane-bound factor to the Golgi apparatus and cleavage by Site-1 protease (S1P) and Site-2 protease (S2P). The released domain of ATF6 enters the nucleus and activates chaperone proteins encoding genes such as Glucose-regulated protein 78 and 94 (GRP78, GRP94) and Calnexin. The active form of ATF6α cooperates with XBP-1 to induce UPR target genes [94,99].

Thus, under exposure to ER stressors, PERK controls translation, whereas IRE1 and ATF6 control activated gene expression. These processes are completed by ER-associated protein degradation (ERAD) which mediates the retro-translocation of unfolded proteins from the ER lumen into the cytosol and promotes degradation by the proteasome. Therefore, ERAD helps other UPR targets such as chaperones by removing misfolded proteins from the ER [100]. An alternative mechanism for degrading severely misfolded proteins is Autophagy which is a catabolic process for the degradation and recycling of cytosolic, long lived or aggregated proteins and excessive or defective organelles. In this process, organelles can be degraded through packaging of ER membranes into autophagosomes and degradation by fusion to vacuoles [101].

All the mechanisms described above aim to reduce the level of ER stress, however, cell death is induced when the primary stimulus is protracted or excessive and the adaptive responses fail to compensate, e.g., it has been reported that 5 mM dithiothreitol (DTT), a reducing agent counteracting disulfide bond formation in protein folding, caused cessation of cellular proliferation (compared with 2.2 mM or 1.5 mM. This indicates a maximal ER stress beyond which cells can no longer compensate effectively [102]. Treating the human embryonic kidney cell line (HEK 293) with Tunicamycin (blocking N-linked glycosylation), has revealed that Xbp-1 mRNA splicing was strongly diminished with prolonged exposure to the drug [103]. Here we are addressing 2 different kinds of agents. DTT can be used to monitor the dose-dependent manner of ER stress response and by tunicamycin can be used to observe the time-dependent manner of ER stress response. It has been shown that after treating the cell line with Tunicamycin and observing the Xbp1, the level was increased after treatment but over time it became depleted and turned back to baseline levels). Thus, apoptosis due to ER stress can also happen in a dose-dependent/ time dependent manner.

### 4.3. ER Stress Outcomes: Survival or Death

A downstream component of ER-stress pathways is C/EBP homologous protein (CHOP, also known as GADD153). CHOP is a transcription factor that promotes cell-cycle arrest and programmed cell death through different intermediate molecules; upregulation of ER oxidase 1α (ERO1 α) or inhibition of gene encoding anti-apoptotic BCL-2 [8]. In parallel, Sustained IRE1 activity, due to ER stressor, leads to activation of c-Jun NH2-terminal kinase (JNK) which activates pro-apoptotic member of the BCL-2 family Bim and inhibit anti-apoptotic Bcl-2, Bcl-XL, and Mcl-1 [96,104,105]. It has been also reported that there is a crosstalk between ER stress pathway and signaling pathway of the mammalian target of rapamycin (mTOR) which is a regulator of protein synthesis and cell growth. This implies an apparent paradox that under particular settings, mTOR, a positive regulator of cell cycle, can also leads to cellular death [106].

To summarize, different sensors are involved in the ER stress response network such as PERK, IRE1 and ATF6 which are the most important components. These proteins lead to decrease in protein biosynthesis and increase in ER folding capacity. These responses can be completed by ER-associated protein degradation and autophagy in presence of aggregated proteins and ineffective organelles. Not being successful to overcome the stressor, in a dose-dependent/time-dependent manner, cells undergo apoptosis and death through activation of pro-apoptotic molecules and inhibition of anti-apoptotic proteins.

### 4.4. ER Stress and Stem Cells

ER stress affects somatic cells as described before. Moreover it can affect cells mediating developmental processes in the embryo and placenta. It is known that cellular protein synthesis has a central role in development of placenta and about 30% of human placental oxygen consumption is demanded by protein synthesis [107]. Under stress conditions the protein synthesis will become compromised to conserve energy. The ER stress in the placenta can be due to maternal malperfusion, fluctuations in intraplacental oxygen concentrations and oxidative stress. Increased ROS can result in the release of calcium from ER by inhibiting the ATP-dependent ion pumps and subsequently Ca2+-dependent chaperone proteins become nonfunctional and protein misfolding and UPR happens. 

It has been shown that by exposure to ER stress-inducing agents, trophoblast-like cell lines, JEG-3 choriocarcinoma cells and Human choriocarcinoma JAR cells, had decreased protein synthesis and suppressed cellular proliferation. Having observed the stress dose-response (ER stress induced by Tunicamycin), increased concentrations were associated with increased levels of CHOP, GRP78 and GRP94 proteins, and splicing of Xbp-1 mRNA [108]. So in this study of trophoblast cell lines, the data showed the placental growth restriction due to protein synthesis inhibition, reduced mTOR signaling due to decreased Akt and apoptosis induction at high levels of stress. 

In a mouse study, examining the embryos at various developmental stages has been done to detect the IRE1 function. The results indicated that the ER stress-dependent IRE1 activation was mainly induced in the placenta but slightly in the embryo. In IRE1α Knockout mice, the cell proliferation of trophoblasts was reduced, resulted in small placentas. ER stress development in IRE1α -/- trophoblasts was poor and PERK, eIF2α, and ATF6 were more activated [109]. This study revealed that IRE1α was activated in the placenta during mouse embryogenesis and the loss of IRE1α, aggravated ER stress in the placenta. Labyrinth layer (a highly vascularized tissue responsible for materno-fetal oxygen and nutrient exchange) was disrupted with a reduction in the internal space of fetal and maternal blood vessels, the level of VEGF-A (vasculogenesis factor of labyrinth which increases by ER stress) in mutant placentas was half of the level in the wild-type placentas. This IRE1α inactivation could lead to embryonic death after 12.5 days of gestation.

As mentioned earlier, GRP78 is a stress-induced Chaperone protein which is activated through ATF6 and binds to the unfolded peptides to promote proper folding. High levels of GRP78 protein are present in the blastocyst, consistent with its physiological role in developing mouse embryos. In a study, Heterozygous Grp78+/- mice had no apparent abnormality in embryos, GRP78 protein level was decreased about 50%, GRP94 was upregulated and the calnexin level and XBP-1 level was similar to the wild-type siblings. In Grp78 -/- embryos, peri-implantation lethality was observed which was due to proliferation defects and a massive increase in apoptosis of ICM (the precursor of embryonic stem cells). Thus it can be concluded that GRP78 involves in different cellular processes including folding of newly synthesized proteins and serves as a sensor of ER stress. Apparently GRP78 is essential for embryonic cell growth and pluripotent cell survival. Both alleles contribute to expression and the lethality of Grp78 -/- embryos indicates that GRP78 function is not compensated by GRP94 or other cellular chaperons [110].

Phosphorylaton of eIF2a phosphorylation leads to translation stop and this also leads to movement of mRNA from ribosomes to stress granules. Stress granules are marked by HuR. The HuR knockout is also a placental lethal and like the IRE1 knockout dies due to failure of the surface of the villous placenta [111]. A cluster of 6 acknowledged stress genes that have villous placental lethal defects were recently reviewed by us [112]. We concluded that there are two types of development. One in which parenchymal function develops from stem cells before the need arises and there is no stress. In a second type need arises before function and stress develops and guides proportional magnitude and timing of onset of parenchymal function. In the case of the villous placenta, IRE1 HuR, HSP90β, p38MAPK, HIF1β, and JunB are required for villous surface development, suggesting that hypoxic stress develops normally and the functions of these genes are needed to respond in mediating stem cell survival and to regulate the differentiation of the stem cells. 

So endoplasmic reticulum is an essential organelle involved in different cellular functions as well as cellular stress response network. ER plays a very critical role in somatic cells, embryonic stem cells and placenta by reduction of protein synthesis and upregulation of chaperones, under stress conditions. In the stem cells, in a time-dependent/dose dependent manner, ER stress can either lead to differentiation to different cell lineages or result in apoptosis of the cells and lethality of embryos.

## 5. Effects of Genotoxic Stress on Stem Cells

We will use one genotoxic stressor, Benzo[a]-pyrene (BaP), to illustrate a large number of stresses that act through genotoxic mechanisms. The particle phase of cigarette smoke contains several polycyclic aromatic hydrocarbons, and in particular BaP [113]. Burnt foods, coal tar, urban diesel fumes and air pollution can also be rich in BaP. BaP can undergo a series of metabolic reactions by cytochrome P4501A (CYP1A1) and produce diol esters (BPDE) that bind to DNA and form mutagenic BPDE-DNA adducts [114] which are known as genotoxic compounds. 

It has been shown that cigarette smoke components are pathogenic for fertility and pregnancy resulting in decreased success rate of *in vitro* fertilization, intrauterine growth restriction, spontaneous abortion and low birth weight [115,116,117]. Accumulation of BPDE-DNA adducts affect placental lineage proliferation and differentiation and crossing the placenta barrier, can be toxic to the developing conceptus [114,118]. In culture, the two-cell stage embryo cannot metabolize BaP, but the blastocyst can [119]. Thus both ESC and TSC in the blastocyst are likely to accumulate genetic errors at a time when very few cells are in each lineage and high fractions of clones can retain errors and other non-genetic stress-induced sequelae. 

BaP significantly induces inhibition of proliferation of human placental cell lines and G2/M cell cycle phase arrest as well as activation of p53, transforming growth factor (TGF)β and suppression of epidermal growth factor receptor (EGFR) [120].

It has been demonstrated that, BaP promoted stress enzyme SAPK and AMPK phosphorylation in a dose and time-dependent manner. Loss of inhibitor of differentiation (ID)2 that maintains TSC potency was also observed. AMPK was necessary for the loss of ID2 induced by BaP [3,121]. Thus embryos treated with BaP *in vitro* activate stress enzyme SAPK and implantation rate reduces significantly. BaP activates MAPK8/9 (SAPK/JNK), p38 mitogen activated protein kinase, Protein kinase AMPK-activated α subunit 1/2 (from the gene *PRKAA1/2* also known as AMPKα1/2) in adult somatic cells and MAPK8/9 in embryos [121,122,123] which are involved in homeostatic and stress responses in embryos and TSC. It has been shown that at 0.25 M concentration of BaP, no significant effect on cell accumulation of TSC was achieved but at 2.5 M cell accumulation was significantly decreased [45]. The lowest BaP dose that reduced cell proliferation and increased cleaved caspase 3 was 0.5 M. The AMPKα1/2 metabolic substrate acetyl CoA carboxylase was also phosphorylated and activated at a threshold dose of 0.5 M BaP. Having studied the time-dependence of AMPKα1/2induction by BaP, each individual dose from 0.25 M to 2.5 M caused significant induction of AMPKα1/2phosphorylation (at Thr172) at 1 hr. The AMPKα1/2 response peaked at 10–30 min and returned to baseline by 4 hr of stimulation. BaP induced a dose-dependent ID2 loss that was maximized at 60% at 2.5 µM, 1 µM and 2.5 µM BaP caused significant loss of ID2 at 24 hr. Like hyperosmolar stress BaP can also induce SAPK-dependent Hand1 upregulation in mouse TSC [124]. Thus BaP-induced AMPKα1/2 Thr172 activation, ID2 protein loss, SAPK activation and Hand1 increase favor TSC differentiation.

## 6. Effect of Hyperosmolar Stress on Stem Cells

Changes in morphology and physiology of the preimplantation embryonic cells developed *in vitro*, displayed the importance of the culture medium quality. In previous studies, it has been illustrated that stress enzymes p38MAPK and SAPK/JNK show different levels of phosphorylation in preimplantation embryos cultured in different media [125].

Hyperosmolar stress has been studied by addition of sorbitol to the medium which allows observation of dose and time-dependent effects on the cells. As mentioned earlier, doses above 200 mM cease TSC accumulation over 24 h of culture. SAPK is functioning in a sigmoidal dose-response manner for sorbitol [5] and along with SAPK activation, increased apoptosis was also observed at higher doses. 

Sorbitol treatment promotes significant HAND1 induction in TSCs which is dose-,time- MAPK8/9 (SAPK/JNK)-dependent [43] so SAPK-dependent differentiation increases. Hand1 mediates induction of PL1 protein markers of first lineage [46]. This is the unique property of stem cells to undergo compensatory differentiation at higher high stress levels that diminish stem cell accumulation [47]. For instance; at low levels of sorbitol, AMPK-dependent phosphorylation and inactivation of ACC occurs which leads to decreased anabolism but at high levels, AMPK-dependent loss of ID2 protein happens and together with SAPK-dependent gain of Hand1, differentiation is induced and cell proliferation and accumulation is suppressed (Figure 2**, **Figure 3, and Figure 4).

In a mouse study, hyperosmolar stress induced ID2 protein loss through AMPKα1/2in TSCs and embryos. By using 200 mM sorbitol, peak level of phosphorylated AMPKα1/2 occurred at 10 min along with rapid loss of ID2. ID2 protein increased 70% after the removal of sorbitol which led to a reversible state of differentiation at lower doses but not at higher doses [42]. Thus at low stress levels cellular adaptation leads to survival, but at high doses, in addition to reduced cell accumulation an organismal survival is induced by differentiation of stem cells to essential lineages. 

## 7. Common and Unique Mechanisms and Outcomes of Various Types of Stress

As mentioned earlier, Organisms are constantly exposed to a variety of stressors such as heat shock or cold treatment, lack of nutrients, low oxygen condition or oxidative stress, ER stress, high osmotic condition, exposure to UV radiation, *etc* [2,3,4,5,6,7,8,9,10,11,12,13,14]. The cellular stress response involves a decrease in normal proteins, RNA, fatty acids and DNA synthesis and increase in a group of stress proteins, highly evolutionarily conserved. Under Oxidative stress, damages in cellular compartments (lipid peroxidation in plasma membrane, protein oxidation and abnormal protein folding, DNA mutation) happen at high concentrations. In this process NF-κB, p38 MAPK and SAPK–JNK pathways become activated and multipotency markers in TSC decrease [50,51,52]. p38MAPK increases expression of the cell cycle inhibitors p16Ink4a and p19Arf and limits the lifespan of stem cells. SAPK mediates a necessary suppression of *polycomb* and alters differentiation of imaginal disc and hematopoietic stem cells in drosophila [70,72]. Oxidative stress activates the Gadd45 promoter in a FoxO-dependent manner which helps with DNA replication and repair [77,78]. Ionizing radiation induces oxidative stress which can be coupled with up regulation of NOX, resulting in genomic instability [81]. 

Protein unfolding and misfolding due to lack of disulfide bonding or glycosylation and ineffective chaperones cause ER stress and PERK (decrease in protein load), IRE1 (encoding the chaperones) and ATF6 (induction of UPR target genes) are the most important components involved in the cellular stress response [88,89,90,91,92]. A downstream component of ER-stress pathways is CHOP that causes cell-cycle arrest. Sustained IRE1 activity activates JNK and BCL-2 family Bim and inhibits anti-apoptotic Bcl-2, Bcl-XL, and Mcl-1, resulting in apoptosis [97,98]. A crosstalk between ER stress pathways and mTOR complex 1 (mTORC1) leads to suppression of Akt signaling, induction of the IRE1–JNK pathway and enhancement of apoptosis [99].

There are some interactions between different forms of stressors and their signaling pathways. For example with regard to oxidative stress and ER stress, oxidative stress induced-ROS can result in disturbance of intracellular Ca2+ homeostasis through the inhibition of ATP-dependent ion pumps and depletion of calcium within the ER lumen causes loss of function of Ca2+-dependent chaperone proteins, accumulation of misfolded proteins within the lumen of endoplasmic reticulum and UPR [51,52,53]. p38MAPK and SAPK are the common enzymes which are shared between many stress response pathways. 

Oct4 (active in embryonic stem cells) is decreased under both oxidative stress and ER stress conditions which results in differentiation of stem cells [59,60,100]. Stem cells of the embryo and placenta have a unique property, known as ''compensatory differentiation'' or ''prioritized differentiation'' [45]. At high doses of stress, they cannot proliferate sufficiently and special kind of differentiation will be induced [46] and stem cells transition from cellular survival to organismal survival. This occurs through induction of early essential differentiated lineages and suppression of later essential differentiated lineages [47].

Another example is benzopyrene stress and hyperosmolar cellular stress response that both induce ID2 protein loss in a proteasome- and AMPKα1/2- dependent manner [42].

## 8. Conclusions

Stress can be induced by many stimuli but shared common outcomes are activation of stress enzymes and, at high enough exposures, diminished expansion of stem cell populations. Three outputs of stress are diminished macromolecular synthesis common to all cell types, tissue and stress-specific adaptive responses (hormesis), and compensatory and prioritized differentiation of stem cells to accomplish the next essential event in development. 

Oxidative, ER, and genotoxic stresses are three major areas of stress response mechanisms and all diminish growth and affect and imbalance stem cell differentiation during development. 

There are important dose-dependent effects of stress. At low doses stress induces cell autonomous stem cell survival responses such as anti-apoptosis and conversion of anabolic to catabolic pathways that include diminished macromolecular synthesis. At lower stress exposures stem cell accumulation is not diminished and there are no effects upon potency loss and gain of differentiation. At higher doses where stem cell accumulation is diminished, stem cells undergo stress-induced differentiation. Fewer cells produce more differentiated product/cell, compensatory differentiation. Thus low stress exposures induce stem cell survival and higher exposures induce organismal survival through stem cell differentiation.

Tightly linked to compensatory differentiation is prioritized differentiation. When a stem cell is potent to produce several different lineages in sequence during development, stress induces the early lineage and suppresses later lineages; prioritized differentiation. Thus prioritized differentiation is a property of stem cells during stress induced gain of differentiation and loss of potency

There are likely to be many mechanisms that regulate compensatory and prioritized differentiation, but we have studied the central role of two kinases that uniquely mediate stress-induced loss of potency and gain of differentiation. In 2-cell stage embryos, blastocysts, and TSC derived from blastocysts, AMPK is necessary and sufficient to mediate stress-induced loss of potency factors such as ID2. Another induced kinase, SAPK, has no role in the stress-induced loss of ID2. However stress-induced upregulation of Hand1 nuclear transcription factor protein and mRNA and Hand1-dependent PL1 require SAPK. Thus one stress-induced kinase mediates potency loss and another mediates gain of differentiation. 

SAPK can respond differently to the same stress dose response if the responder is a somatic cell or a stem cell (S- or U-shaped response to an O_2_ dose response, respectively). However, SAPK function in stem cells is to increase the first lineage (e.g. Hand1) in response to stimuli that produce S-shaped (hyperosmolar) or U-shaped (incorrect O_2_ levels) responses. In the case of the U-shaped response to incorrect SAPK increases Hand1 in proportion to the departure from the optimal O_2 _for stemness of TSC. We anticipate that SAPK or other similar stress enzymes will mediate suppression of later lineages in response to multiple stressors. It is possible that prioritized differentiation, and its stress enzyme-mediated regulation, is intrinsic to later phases of embryonic development, cancer stem cell function, and adult stem cell function.
